# Spatiotemporal Variability and Influencing Factors of Aerosol Optical Depth over the Pan Yangtze River Delta during the 2014–2017 Period

**DOI:** 10.3390/ijerph16193522

**Published:** 2019-09-20

**Authors:** Liang Cheng, Long Li, Longqian Chen, Sai Hu, Lina Yuan, Yunqiang Liu, Yifan Cui, Ting Zhang

**Affiliations:** 1 School of Environmental Science and Spatial Informatics, China University of Mining and Technology, Daxue Road 1, Xuzhou 221116, China; liang.cheng@cumt.edu.cn (L.C.); long.li@cumt.edu.cn (L.L.); saihu@cumt.edu.cn (S.H.); lnyuan@cumt.edu.cn (L.Y.); yunqiang.liu@cumt.edu.cn (Y.L.); yifan.cui@cumt.edu.cn (Y.C.); tingzhang@cumt.edu.cn (T.Z.); 2 Engineering Research Center of Ministry of Education for Mine Ecological Restoration, China University of Mining and Technology, Daxue Road 1, Xuzhou 221116, China; 3 College of Yingdong Agricultural Science and Engineering, Shaoguan University, Daxue Road 26, Shaoguan 512005, China; 4 Department of Geography, Earth System Science, Vrije Universiteit Brussel, Pleinlaan 2, Brussels 1050, Belgium

**Keywords:** aerosol optical depth (AOD), Pan Yangtze River Delta, MODIS, gap-filling, geographical detector method, topography

## Abstract

Large amounts of aerosol particles suspended in the atmosphere pose a serious challenge to the climate and human health. In this study, we produced a dataset through merging the Moderate Resolution Imaging Spectrometers (MODIS) Collection 6.1 3-km resolution Dark Target aerosol optical depth (DT AOD) with the 10-km resolution Deep Blue aerosol optical depth (DB AOD) data by linear regression and made use of it to unravel the spatiotemporal characteristics of aerosols over the Pan Yangtze River Delta (PYRD) region from 2014 to 2017. Then, the geographical detector method and multiple linear regression analysis were employed to investigate the contributions of influencing factors. Results indicate that: (1) compared to the original Terra DT and Aqua DT AOD data, the average daily spatial coverage of the merged AOD data increased by 94% and 132%, respectively; (2) the values of four-year average AOD were high in the north-east and low in the south-west of the PYRD; (3) the annual average AOD showed a decreasing trend from 2014 to 2017 while the seasonal average AOD reached its maximum in spring; and that (4) Digital Elevation Model (DEM) and slope contributed most to the spatial distribution of AOD, followed by precipitation and population density. Our study highlights the spatiotemporal variability of aerosol optical depth and the contributions of different factors over this large geographical area in the four-year period, and can, therefore, provide useful insights into the air pollution control for decision makers.

## 1. Introduction 

Aerosols, the liquid or solid particulate matter suspended in the atmosphere [1], have both natural and anthropogenic sources, such as volcanic eruptions, sand, dust, fossil fuel combustion, and industrial and traffic emissions [2,3,4]. By absorbing and scattering solar radiation and perturbing the hydrological cycle, aerosols have a crucial effect on regional and global climate change [5,6,7]. In addition, numerous aerosol particles contribute to increased levels of haze and lead to low visibility [8,9,10,11]. Furthermore, epidemiological studies worldwide have associated aerosol particles, especially coarse particles (with an aerodynamic diameter ≤ 10 μm; PM_10_) and fine particles (with an aerodynamic diameter ≤ 2.5 μm; PM_2.5_) with adverse health outcomes, including increased mortality and morbidity of cardiovascular and respiratory diseases [12,13]. Aerosol optical depth (AOD), which is defined as the integral of the extinction coefficient of aerosol in the vertical direction, indicates the attenuation of the light induced by aerosols and the degree of atmospheric pollution [14,15]. As such, AOD is often used to monitor air quality and to evaluate the aerosol effect on climate [16].

Ground-based observations networks such as the Aerosol Robotic Network (AERONET) can provide accurate time-series AOD observations at different sites around the world [17,18]. Due to the limited number of observation sites, it is however difficult to produce AOD data that cover large geographical areas [16,19]. Satellite-based observations, such as Moderate Resolution Imaging Spectrometers (MODIS) aerosol optical depth (AOD) products, have recently been proved useful to guarantee the spatiotemporal continuity of AOD observations [16,20]. The MODIS AOD products provide daily operational retrievals over land based on Dark Target (DT) or Deep Blue (DB) algorithm [21,22]—the DT algorithm was developed to retrieve AOD only over dark surfaces (e.g., water and dense vegetation) while the DB algorithm worked both over dark surfaces and bright surfaces (e.g., arid, semiarid, and urban areas) [23,24]. In 2017, the MODIS Collection 6.1 (C6.1) AOD products have been released, consisting of 10-km resolution DT, DB, and merged DT and DB (DTB) datasets, and a 3-km resolution DT AOD dataset [25,26]. Compared with the 10-km AOD, the 3-km product resolves aerosol plumes and provides better aerosol gradients [27,28]. Nevertheless, AOD may have no data over bright-reflecting regions because of the limited applicability of the DT algorithm [29,30]. In addition, clouds, high surface reflectance, and retrieval errors can also frequently cause a large amount of missing data in AOD datasets [31,32]. The data gaps (i.e., the no-data areas) in DT AOD can disable a spatiotemporal characteristics analysis of aerosols and constrain air quality monitoring.

To address the issue of missing AOD data, several gap-filling methods have been proposed. One method is using the Kriging interpolation to estimate the missing data based on the spatial autocorrelation [33] or spatiotemporal autocorrelation [34] of AOD values. Another method is developing statistical models for AOD imputation with other parameters (e.g., PM_2.5_ or cloud fraction, elevation and some meteorological parameters) [32,35]. In addition, merging multi-source AOD datasets was also proved useful in filling AOD data gaps, taking advantage of the different spatial coverage of multiple AOD datasets [25]. Commonly used merging techniques include the maximum likelihood estimate [36,37], the inverse variance weighting [38], and linear regression [22,31,39]. Among them, the linear regression method has less calculation work while showing an acceptable performance. For example, He and Huang established linear regression models to merge MODIS 3-km DT AOD and 10-km DB AOD data, increasing data availability temporally by 10–50% over the original 3-km Aqua/Terra data for China [22]. However, the accuracy of merged AOD data was only validated for two AERONET AOD sites within and around Beijing. Due to the large area of China, the daily relationships between different AOD data (i.e., Terra and Aqua AOD or DT and DB AOD) are likely to vary with space and scale, and this should be tested on a regional scale. As the first attempt, we adopted the method proposed by He and Huang to merge AOD data on a regional scale, filling missing values in the 3-km DT AOD.

Variation in the spatial distribution of AOD results from a combination of multiple factors, such as topography, meteorology, vegetation, and socioeconomic factors [6,19,40,41,42]. Topography has been reported to be correlated negatively with the spatial pattern of AOD in several studies because of its strong relation with aerosol emissions and particle accumulation [19,41,42,43]. Meteorological variables, such as precipitation [19,44,45,46], wind speed [45,47,48], temperature [46,49,50,51], relative humidity [45,52] and planetary boundary layer height (PBLH) [53,54] play important roles in the diffusion, dilution, and accumulation of aerosol particles. The effect of vegetation on AOD varies in different areas of China. For example, the AOD-NDVI relation was observed positive in areas close to the Heihe-Tengchong Line while negative in the provinces of Zhejiang, Hubei, and Guangdong [42,44,50]. Previous studies have reported the impacts of socioeconomic factors on AOD, e.g., gross domestic product (GDP) and population density, both found positively correlated to AOD [40,45,50]. Although a variety of approaches, including correlation analysis [40], linear regression [50], and geographically weighted regression [42] were used to identify the controlling factors of AOD, they either ignore the spatial characteristics of those factors and AOD or tend to be restricted by collinearity among those factors [55,56]. The geographical detector method, proposed by Wang et al. [57], has been proved effective in quantifying the contributions of factors to various geographical phenomena [56,58,59]. This method can reveal the influencing factors based on the concept of stratified spatial heterogeneity without linear assumptions [57]. Additionally, the geographical detector method is not limited by collinearity (i.e., any potential factors can be included in the analysis without having to consider the problem of collinearity) [56,59,60]. However, there were few studies on geographical detector method for AOD. The present study used the geographical detector method to explore the relationships between AOD distribution and multiple factors. Meanwhile, since the geographical detector method is not able to reveal the impact direction (i.e., negative or positive) of each factor, multiple linear regression analysis was employed to investigate the nature of the impact [56,59].

The Pan Yangtze River Delta region (PYRD) [61,62], lying in the intersection of the “Belt and Road” and the “Yangtze River Economic Belt” in China [63], has long suffered severe air pollution due to its rapid economic development and urbanization [22]. However, previous studies on aerosol pollution were mainly conducted for the Yangtze River Delta region (YRD) [32,64,65] with few studies focusing on Anhui [52,66,67], a province is adjacent to the YRD with a high concentration of particulate matter [22]. These studies usually mapped air pollution characteristics at 10-km spatial resolution or even lower, hardly capturing regional-scale pollution variability [16,66]. Additionally, the quantitative determination of the contribution of each potential factor to AOD has been little studied. 

Therefore, this study attempts to analyze the spatiotemporal characteristics of AOD over the PYRD with a merged fine-resolution AOD dataset and then determine the effects of factors on AOD distribution. Specific objectives are: (1) to improve the spatial and temporal coverage of MODIS AOD data over the PYRD during 2014–2017 by merging four MODIS AOD datasets, namely Terra 3-km DT AOD, Aqua 3-km DT AOD, Terra 10-km DB AOD, and Aqua 10-km DB AOD; (2) to characterize the spatial pattern and temporal variation of AOD over the PYRD and its four parts; and (3) to reveal the contributions of topography, meteorology, vegetation, and socioeconomic factors to the spatial variations of AOD over the PYRD through the geographical detector method and multiple linear regression method.

## 2. Study Area

**Figure 1:**
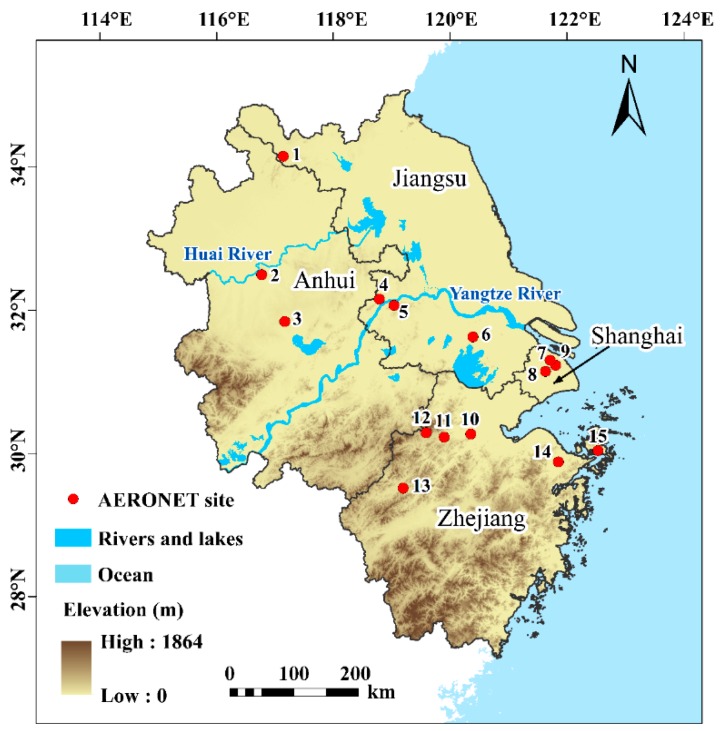
The geographical locations of the PYRD and AERONET sites. Details of these sites are given in Table 2. Data from these sites were used for calibration and validation in Section 3.2.1 and Section 3.2.2.

Located in the mid-east China (27°12’ N~35°20’ N, 114°54’ E~123°10’ E), the Pan Yangtze River Delta region (PYRD) consists of the provinces of Anhui, Jiangsu, Zhejiang and the provincial-level municipality of Shanghai, with an area of approximately 357,282 km^2^ (Figure 1). With low elevations in the northeast and high in the southwest, the PYRD is characterized by diverse geomorphological features including plains, tablelands, hills and mountains [68]. The plains are mainly distributed in Jiangsu, Shanghai, and north Anhui, while most of the hills and mountains are scattered in Zhejiang and southeast, and southwest of Anhui. Divided by the Huai River, the PYRD has a subtropical monsoon climate in the south with hot, rainy summers and mild winters but a temperate monsoon climate in the north with hot, rainy summers and cold, dry winters.

As one of the most densely populated and economically developed regions in China, the PYRD has a population of some 223 million people and generated a GDP (gross domestic product) of 19.53 trillion CNY (Chinese yuan) in 2017, accounting for approximately 16.08% and 23.05% of China’s total population and GDP, respectively. However, the population density and GDP per capita varied across the PYRD, being highest in Shanghai (3813 people/km^2^ and 126,687 CNY) and lowest in Anhui (448 people/km^2^ and 43,194 CNY). Despite its important role in China’s economic growth, the PYRD has experienced severe haze pollution since 2013.

According to the China Statistical Yearbook on Environment 2015 [69], the annual average concentrations of PM_2.5_ and PM_10_ in 18 environmental key cities in the PYRD in 2014 ranged from 46 µg m^−3^ to 83 µg m^-3^ and 71 µg m^−3^ to 124 µg m^−3^, respectively, all exceeding the Chinese Ambient Air Quality Grade II standard (PM_2.5_: 35 µg m^−3^, PM_10_: 70 µg m^−3^) [70]. To mitigate these serious levels of air pollution, China’s State Council issued the National Action Plan for Air Pollution Prevention and Control in September 2013, followed by the regional rule for the implementation of National Action Plan in the PYRD jointly released by governments of Anhui, Jiangsu, Zhejiang, and Shanghai in January 2014 [67]. There is therefore an urgent need to examine the effect of such regulations and to further explore the influencing mechanism of the factors contributing to the AOD.

## 3. Data and Methods

### 3.1. Data

#### 3.1.1. MODIS AOD Data 

MODIS sensors onboard Terra and Aqua satellites, both launched by the U.S. National Aeronautics and Space Administration (NASA), provide daily AOD measurements [71]. Terra and Aqua satellites cross the equator separately at approximately 10:30 a.m. and 1:30 p.m. local solar time [31]. MODIS Collection 6.1 (C6.1) Level 2 aerosol products from 1st January 2005 to 30th December 2017 covering the PYRD were obtained from the website of Level 1 and Atmosphere Archive and Distribution System (LAADS) [72]. The downloaded MODIS AOD data products consist of 3-km DT AOD and 10-km DB AOD from both Terra and Aqua satellites. The expected error (EE), which represents a one-standard deviation confidence interval around the retrieved AOD (i.e., about 68% of points should fall within ±EE from the AERONET AOD), is ±(0.05 + 20%) for the 3-km DT retrievals over land [27,73]. For the 10-km DB retrievals, the EE is defined relative to DB-retrieved AOD rather than to AERONET AOD, is approximately ±(0.03 + 20%) on average [29,73]. In this study, only those AOD retrievals at 550 nm with the recommended quality assurance (QA) for the DT (QA = 3) and DB (QA ≥ 2) were selected [26]. Therefore, the DT and DB high-quality retrievals were obtained from the Scientific Data Set (SDS) “Optical_Depth_Land_and_Ocean” in the 3-km DT products and “Deep_Blue_Aerosol_Optical_ Depth_550_Land_Best Estimate” in the 10-km DB products, respectively. Due to their relatively high spatial resolution, the 3-km DT AOD datasets were selected as the main source to illustrate the spatiotemporal characteristics of AOD over the PYRD [27]. However, the DT algorithm does not perform well over bright surfaces. To fill the gaps left by the 3-km DT AOD, the 10-km DB AOD datasets were used as supplementary source and merged to the 3-km DT AOD datasets because of their better performance over bright targets [22]. MODIS AOD data derived from 2005 to 2013 were utilized for AOD calibration (Section 3.2.1). Data from 2014 to 2017 were calibrated and then employed for spatiotemporal characteristics and influencing factors analysis of AOD. Table 1 provides a summary of MODIS AOD data products used in this study.

#### 3.1.2. AERONET AOD Data 

In order to validate MODIS AOD values, the high-accuracy ground-based aerosol measurements from 2005 to 2017 were obtained from the Aerosol Robotic Network (AERONET) [74], a global aerosol observation network recording AOD observations by CE-318 Solar Photometer every 15 min with an uncertainty of ~0.01–0.02 under cloud-free conditions [75,76,77,78]. The AERONET offers three levels of AOD data, Level 1.0 without strict quality checks, Level 1.5 with cloud screening checks, and Level 2.0 with rigorous quality checks [34]. Due to the accuracy and volume of data, the Level 1.5 AERONET AOD data at 15 sites (Table 2) in the region were chosen for validation [79,80].

#### 3.1.3. Auxiliary Data

Ten potential factors affecting the spatial distribution of AOD were selected from four categories, namely topography, meteorology, vegetation, and socioeconomics. To derive these factors, multi-sources were collected.

The 90-m resolution Shuttle Radar Topography Mission Digital Elevation Model (SRTM DEM) data were freely obtained from the website of Consultative Group for International Agriculture Research Consortium for Spatial Information (CGIAR-CSI) [81] and used to provide DEM and slope of the study area. Monthly meteorological dataset observed at 87 observation stations within and around the study area from 2014 to 2017 were downloaded from the China Meteorological Data Service Center (CMDC) [82]. The dataset provides meteorological information including precipitation (PREC), average wind speed (AWS), average temperature (ATEM) and average relative humidity (ARH). As planetary boundary layer height (PBLH) data were not available at this website, the monthly PBLH data from 2014 to 2017 were collected from the European Center for Medium-Range Weather Forecasts [83], with a horizontal resolution of 0.125° × 0.125°. The normalized difference vegetation index (NDVI) data from 2014–2017, representing the vegetation coverage of the study area, were acquired from the Data Center for Resources and Environmental Sciences, Chinese Academy of Sciences (RESDC) [84]. RESDC provides seasonal NDVI values and annual NDVI values at 1-km resolution [41]. From this website, we also obtained the 1-km resolution annual gross domestic product (GDP) and population density (POP) data of the study area in 2015 to reflect the anthropogenic emissions of pollutants from 2014 to 2017. 

### 3.2. Methodology

**Figure 2:**
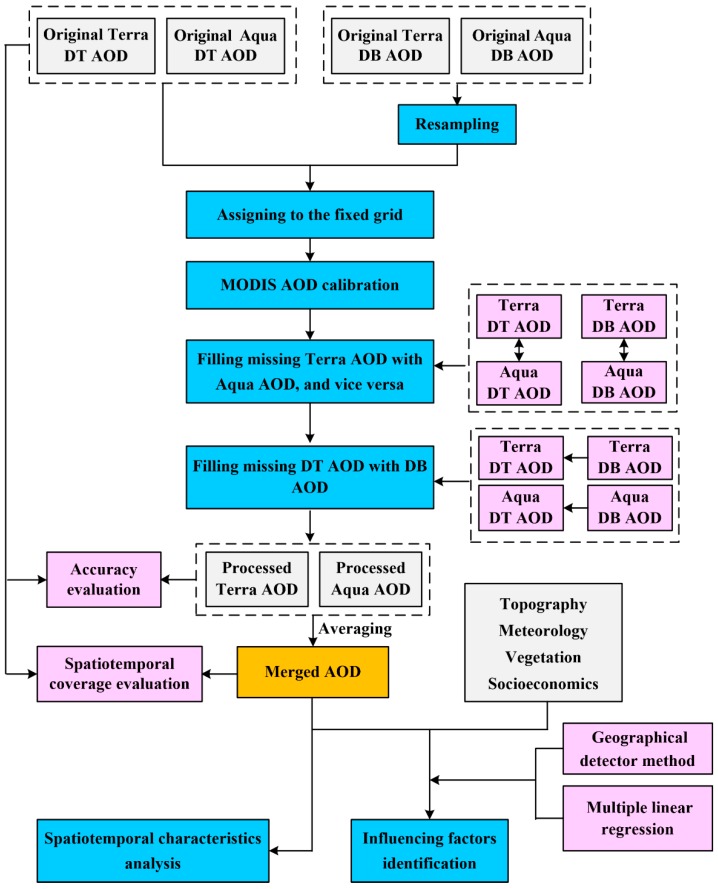
The framework of the study procedure.

The general process of this study is shown in Figure 2. A fixed 3 × 3 km grid (40,801 cells in total) was first created in the extent of the PYRD, as the MODIS AOD pixel centroids varied from day to day [71]. To be consistent with DT AOD, the 10-km DB AOD data were resampled to 3-km resolution using the nearest neighbor method in ENVI 5.3 (Exelis Visual Information Solutions, Boulder, CO, USA), as shown in previous studies [25,85], based on the assumption that the variability of DB AOD data is small within the 10 × 10 km grid. To check whether the other resampling methods can improve the accuracy of the resampled AOD data, bilinear interpolation and cubic convolution were also used for AOD interpolation and the validation results show the nearest neighbor method outperformed the other two methods (Table A1 in Appendix A). Next, daily AOD pixel values from the four datasets (Terra 3-km DT AOD, Aqua 3-km DT AOD, Terra 3-km DB AOD, and Aqua 3-km DB AOD) were matched to the 3-km grid cells whose centroids were within a given grid cell [32], using the extraction tool in ArcGIS 10.2 (Esri, Redlands, CA, USA). To fill AOD data gaps, DB AOD and DT AOD data were merged and then the merged effect was evaluated. After that, the merged AOD data were utilized for spatiotemporal analysis and identification of influencing factors. 

#### 3.2.1. MODIS AOD Merging

To fill AOD data gaps, a four-step merging approach was utilized to merge DT AOD and DB AOD data, following the method proposed by He and Huang [22]:

Step 1: Calibrating the MODIS AOD data. To reduce the systematic bias in satellite-retrieved AOD values, simple linear regression relationships between AERONET AOD and MODIS AOD from 2005 to 2013 were developed to calibrate the MODIS AOD data during 2014–2017 period [21,30]. Since the relationship between AERONET AOD and MODIS AOD varied by season and AOD dataset, linear relationship analysis was conducted for each of the four MODIS AOD dataset (i.e., Terra/Aqua 3-km DT AOD and Terra/Aqua 10-km DB AOD) and each season, separately (Table A2 in Appendix A) [22,27,39]. The seasons were defined in this study as spring (March, April, and May), summer (June, July, and August), autumn (September, October, and November), and winter (December, January, and February).

Step 2: Filling missing Terra AOD data with Aqua AOD values, and vice versa. Owing to the contrasting crossing times, the AOD data retrieved from the two satellites (i.e., Terra and Aqua) differ in spatial coverage [21,22,34,42]. Therefore, for AOD datasets retrieved by the same algorithm (i.e., DT or DB AOD), a simple linear regression model between Terra and Aqua values were developed for each day to fill the missing Terra/Aqua AOD data with the present one (e.g., predicting the missing Aqua DT AOD with the present Terra DT AOD, and vice versa) [86]. It is notable that extra biases may be generated in this step, due to the changing PBLH and aerosol concentration between two satellite overpass times. We acknowledge the limitation of this approach. However, it is a common and effective practice to predict missing AOD values for Terra or Aqua AOD [22,31,86]. Additionally, Pearson correlation coefficients also indicate that there were high correlations between AERONET AOD values at the two satellite passing times (Table A3 in Appendix A).

Step 3: Filling missing DT AOD data with DB AOD values. To fully exploit the retrievals of both DT and DB algorithms, linear regression relationships between daily DT and DB AOD values were established and used to predict values in the no-data pixels in DT AOD when only DB AOD is present [22,42]. After this step, two gap-filled AOD datasets were generated, called as processed Terra AOD and processed Aqua AOD respectively.

Step 4: Averaging the daily Terra AOD and Aqua AOD values. The average of the daily processed Terra AOD and Aqua AOD values (both overserved and predicted values) were calculated and considered as the final daily AOD data (merged AOD hereafter) [22,31,39,42]. 

#### 3.2.2. Merged AOD Evaluation

Ten-fold cross-validation (CV) method was used to evaluate the performance of linear regression models. The original data were randomly divided into 10 groups. From the 10 groups, nine groups of data were used as training data for developing the model, and the remaining group was used to test its predictions. This step was then repeated 10 times until every fold was tested. The commonly used statistical metrics, including root mean squared error (RMSE), relative prediction error (RPE), and the coefficient of determination (R^2^) were used to measure the predictive performance of the models [32,87]. The standard deviation (σ) of the predicted AOD values in each step (i.e., Step 2 and Step 3 in Section 3.2.1) were also calculated.

To examine the performance of the merging operation, accuracy comparison was conducted between the original Terra/Aqua DT AOD and processed Terra/Aqua AOD data, because the merged AOD data cannot be collocated with the AERONET AOD measurements in time [22]. In addition to the accuracy comparison, we also examined if the coverage of the merged AOD data higher than that of original Terra DT AOD and Aqua DT AOD data. Here, the coverage includes daily spatial coverage (denotes the ratio of AOD available pixels of all the pixels for each day) and pixel-level temporal coverage (denotes the ratio of the AOD available days of the whole study period for each pixel) [21].

Linear fitting of MODIS AOD with corresponding AERONET AOD data was used to validate the accuracy of MODIS AOD. Since AERONET AOD are point measurements at 15-minute intervals while MODIS AOD are instantons data when the satellites overpass, MODIS AOD retrievals and AERONET measurements cannot be compared directly and need to be matched in space and time. Thus, following the method of previous studies [77,80], the MODIS AOD retrievals within 5 × 5 pixels (i.e., 15 × 15 km) centered over the AERONET sites were averaged and then collocated with the mean values of the AERONET AOD measurements within 30 min of the time when MODIS passes over. Note that the MODIS AOD were retrieved at 550 nm while AERONET does not provide AOD data at 550 nm, AERONET AOD at 550 nm was derived by interpolating the AERONET AOD values at 440 nm and 675 nm with Equation (1) and Equation (2) [34,80]:

(1) $$\alpha_{\lambda_{1}\sim \lambda_{2}}=-\frac{ln\left(\tau_{\lambda_{1}}/\tau_{\lambda_{2}}\right)}{ln\left(\lambda_{1}/\lambda_{2}\right)},$$

(2) $$\tau_{\lambda_{3}}=\tau_{\lambda_{2}}\times {\left(\frac{\lambda_{3}}{\lambda_{2}}\right)}^{-\alpha_{\lambda_{1}\sim \lambda_{2}}},$$

where $\tau_{\lambda_{1}}$, $\tau_{\lambda_{2}}$ are AOD at the two closest bands $\lambda_{1}$ (440 nm) and $\lambda_{2}$ (675 nm), respectively, $\tau_{\lambda_{3}}$ are AOD at 550 nm.

Several statistical indicators were selected for comparison of values between MODIS AOD and AERONET AOD, such as the number of matched MODIS AOD and AERONET AOD pairs (N), correlation efficient (R), root mean squared error (RMSE), and the percentage retrievals within the expected error (EE, ±(0.05 + 20%$\tau_{A}$), where $\tau_{A}$ is the AERONET AOD) envelope [23,88]. 

#### 3.2.3. Data Integration

For the analysis of spatiotemporal variability and influencing factors, the daily merged AOD (Section 3.2.1) and auxiliary data (Section 3.1.3) were further processed. The seasonally and annually averaged AOD were derived by averaging the daily merged AOD. The monthly meteorological data (i.e., PREC, AWS, ATEM, ARH and PBLH) and NDVI data (both seasonal and annual data) [41] were converted to four-year seasonal and annual average data. After that step, the meteorological data (i.e., PREC, AWS, ATEM, ARH) at each site were interpolated to 3-km continuous raster data by the inverse distance weight interpolation method [38]. PBLH data were resampled to 3 × 3 km grid cell by bilinear interpolation [89]. For the other variables (i.e., DEM, SLP, NDVI, GDP and POP), the corresponding values of the pixels fell in each grid cell were averaged separately to match the fixed 3 × 3 km grid [90].

#### 3.2.4. Influencing Factors Identification

To identify the intensity and directions of the impacts of factors on AOD, geographical detector method and multiple linear regression analysis were used. Based on the concept of spatial stratification heterogeneity—which refers to a geographical phenomenon that the observations are homogeneous within each stratum rather than between strata, the geographical detector method can quantify the contributions of influencing factors [91]. The philosophy of this method is that if an independent variable X (the factor) takes on a similar spatial distribution to that of the dependent variable Y (AOD), there is a direct or indirect relationship between the variable X and dependent variable Y [57]. The geographical detector method examines if an independent variable X takes on a similar spatial distribution with the dependent variable Y and measures the association between Y and X by the power of determinant (q) [91]. Here, the power of determinant (q) indicates how much X contributes to the spatial stratification heterogeneity of Y, or how much Y is interpreted by X [92].

Specifically, a study area is composed of N units, and the AOD in each unit is denoted as $Y_{i}\;\left(1\leq i\leq N\right)$. The factor (X) layer is stratified into h=1, …, L stratum according to the spatial heterogeneity first, and then the AOD (Y) layer is divided into L stratum also by overlaying the Y layer and X layer. Stratum h has $N_{h}$ units and $N=\overset{L}{\underset{h=1}{\sum }}N_{h}$. In stratum h, the AOD in each unit is denoted as $Y_{hi}\;\left(1\leq hi\leq N_{h}\right)$. For the whole study area, the mean value and variance of AOD are $\bar{Y}=\left(1/N\right)\overset{N}{\underset{i=1}{\sum }}Y_{i}$ and $\sigma^{2}=\left(1/N\right)\overset{N}{\underset{i=1}{\sum }}{\left(Y_{i}-\bar{Y}\right)}^{2}$, respectively. For stratum h, the mean value and variance of AOD are $\bar{Y_{h}}=\left(1/N_{h}\right)\overset{N_{h}}{\underset{i=1}{\sum }}Y_{hi}$ and $\sigma_{h}^{2}=\left(1/N_{h}\right)\overset{N_{h}}{\underset{i=1}{\sum }}{\left(Y_{hi}-\bar{Y_{h}}\right)}^{2}$, respectively. The power of determinant (q) of X to Y can be expressed as [91]:

(3) $$q=1-\frac{\sum_{h=1}^{L}N_{h}\sigma_{h}^{2}}{N\sigma^{2}}=1-\frac{SSW}{SST},$$

(4) $$SSW=\overset{L}{\underset{h=1}{\sum }}N_{h}\sigma_{h}^{2},$$

(5) $$SST=N\sigma^{2},$$

where SSW is the within sum of the squares; SST is the total sum of the squares. If SSW is less than SST, spatially stratified heterogeneity exists. 

Usually, q∈[0,1]. If q=1, it means that X can explain 100% of Y; If q=0, there is no association between X and Y. A larger q value indicates a greater influence of X on Y. Following the threshold set by Tang et al. [93], we considered a factor had an important contribution to AOD when the q value of this factor approaches 0.2. 

In addition, as the geographical detector method can only measure the explanatory power of factors, but cannot reveal the nature of the effect (i.e., negative or positive) [56,59], the multiple linear regression was performed as a supplement to identify such information [56]. To avoid the collinearity issue, Pearson correlation coefficients among influencing factors were used to select the variables for model building [21,45,94]. The positive or negative regression coefficients in the multiple linear regression model indicate that the impact of some factor on AOD is positive or negative. The variation inflation factor (VIF) is used to measure the multicollinearity among multiple regression variables [95], if VIF is less than 3, it indicates that there is no collinearity in the regression model. The geographical detector method and multiple linear regression analysis were conducted using GeoDetector [91] and IBM SPSS Statistics 20.0 (SPSS Inc., Chicago, IL, USA), respectively.

## 4. Results

### 4.1. Evaluation of the Merged AOD

#### 4.1.1. Validation of the Merged AOD 

The cross-validation results (Table A4 in Appendix A) demonstrate the good predictive performance of the linear regression models, with RMSE ranging from 0.09 to 0.14, RPE ranging from 20.8% to 29.6% and R^2^ ranging from 0.82 to 0.88 for the years and the four-year period. The σ of the predicted AOD values range from 0.0011 to 0.0110 for the four-year period of 2014–2017.

**Figure 3:**
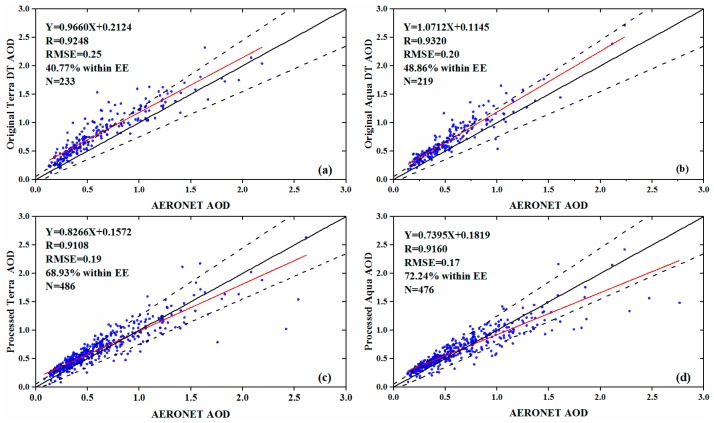
Comparison between AERONET AOD and original Terra/Aqua DT AOD and processed Terra/Aqua AOD data: (**a**) AERONET AOD vs. original Terra DT AOD; (**b**) AERONET AOD vs. original Aqua DT AOD; (**c**) AERONET AOD vs. processed Terra AOD; (**d**) AERONET AOD vs. processed Aqua AOD. The dashed, black, and red solid lines are the EE line, 1:1 line, and fitting line of linear regression respectively.

Figure 3 shows the comparison between the AERONET AOD data from 2014 to 2017 against the original Terra/Aqua DT AOD data and the processed Terra/Aqua AOD data. Overall, the processed Terra/Aqua AOD data approximated the AERONET better than the original Terra/Aqua DT AOD data. After merging, there are 486 and 476 pairs of matched data for processed Terra and Aqua AOD with AERONET AOD, respectively. For the processed Terra AOD, the RMSE decreased from 0.25 to 0.19, and the percentage of retrievals within the EE increased from 40.77% to 68.93%. For the processed Aqua AOD, the RMSE decreased from 0.20 to 0.17, and the percentage of retrievals within the EE increased from 48.86% to 72.24%. However, the R-value of the processed AOD showed a slight decrease, from 0.9248 to 0.9108 for Terra and from 0.9320 to 0.9160 for Aqua. 

#### 4.1.2. Assessment of the Spatiotemporal Coverage of the Merged AOD

**Figure 4:**
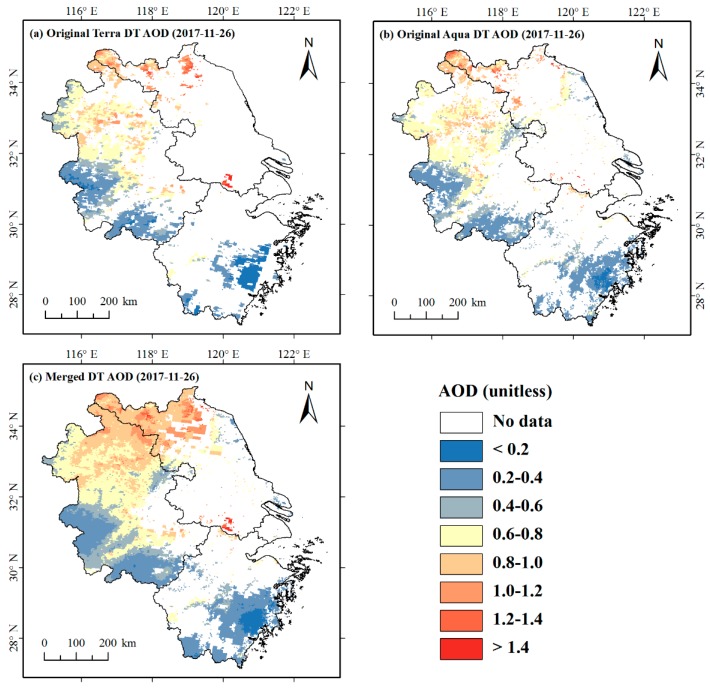
The spatial distribution of (**a**) original Terra DT AOD, (**b**) original Aqua DT AOD, and (**c**) merged AOD on November 26, 2017.

**Figure A1:**
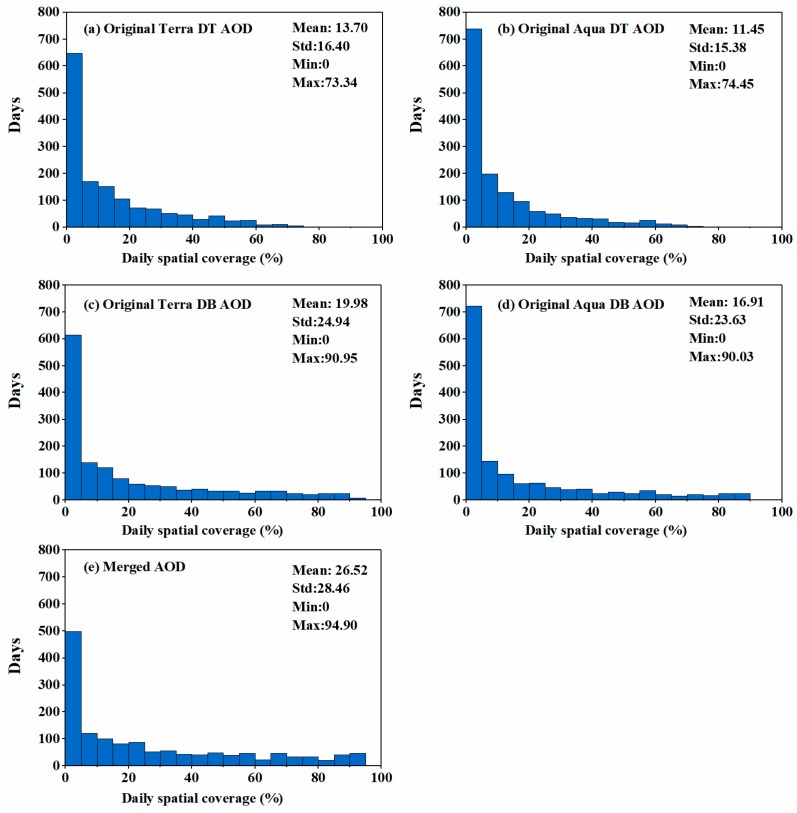
The histograms of the daily spatial coverage of (**a**) original Terra DT AOD; (**b**) original Aqua DT AOD; (**c**) original Terra DB AOD; (**d**) original Aqua DB AOD; and (**e**) merged AOD.

The objective of this present study is to improve the coverage of the DT AOD data with the available DB AOD retrievals. To evaluate the merging effect, the daily spatial coverage of the original Terra DT AOD, Aqua DT AOD and the merged AOD data were compared (Figure A1 in Appendix A). After merging, the average daily spatial coverage was greatly increased, by 94% and 132% compared to the original Terra DT AOD and Aqua DT AOD. For the original Terra DT AOD and Aqua DT AOD data, there were 71 and 64 days with spatial coverage of more than 50%, while for the merged AOD data, there were 323 days with spatial coverage of more than 50%. Figure 4 illustrates the spatial distribution of the original Terra DT AOD, Aqua DT AOD and the merged AOD data on 26 November 2017. For the data of this date, the spatial coverage of the merged AOD was 51.42%, much higher than that of the original Terra DT AOD (25.61%) and Aqua DT AOD (28.54%).

**Figure 5:**
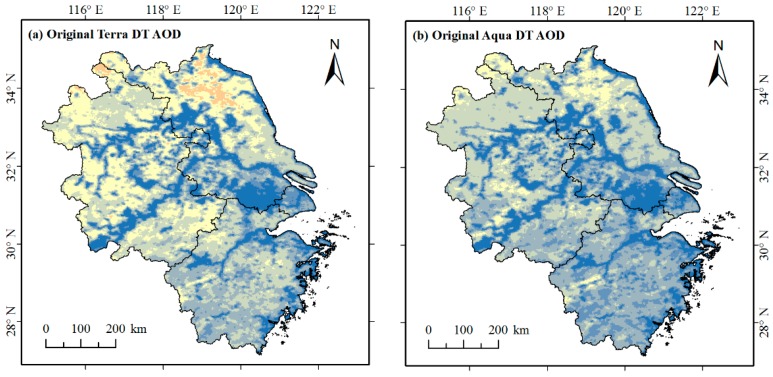

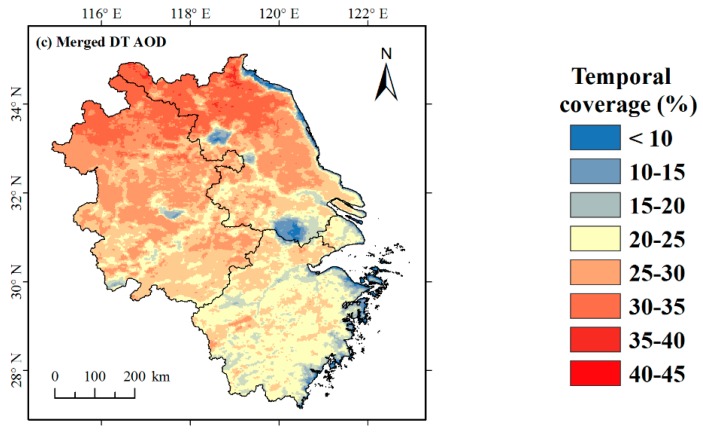
The spatial distribution of temporal coverage (pixel-level) of (**a**) original Terra DT AOD, (**b**) original Aqua DT AOD, and (**c**) merged AOD.

The temporal coverage was also compared as shown in Figure 5. It is clear that the temporal coverage of the merged AOD data was higher than the original. From 2014 to 2017, there were 1451 days for which both Terra and Aqua AOD data were available (there were 10 days for which only Terra AOD data were available and we discarded them). The temporal coverage (pixel-level) of the original Terra DT AOD and Aqua DT AOD data ranged from 0 to 29.08% and from 0 to 25.36% (Figure 5a,b), respectively. After merging, the temporal coverage of AOD data for most of the PYRD ranged from 20% to 40% (Figure 5c). 

The temporal coverage of the merged AOD data varied from area to area (Figure 5c). While more days were available in north Jiangsu and north Anhui with the percentage of availability mostly from 25% to 40%, fewer days were available in Shanghai and the most of Zhejiang with the percentage of availability mostly ranged from 20% to 25%. 

### 4.2. Spatiotemporal Characteristics of AOD

#### 4.2.1. Spatial Variations of AOD 

**Figure 6:**
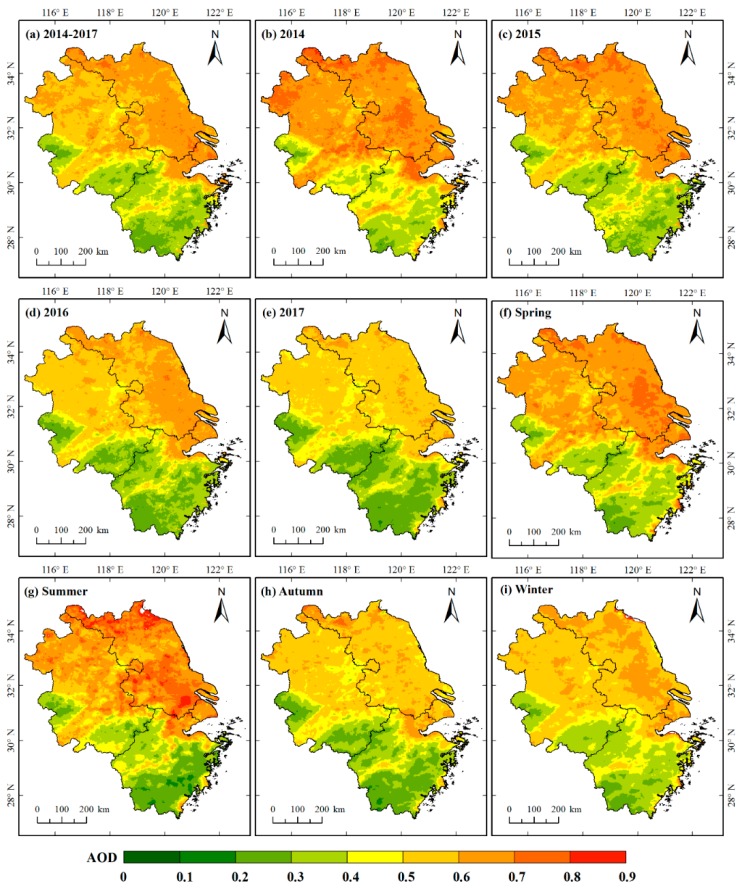
The spatial distribution of (**a**) four-year average AOD, (**b**–**e**) annual average AOD, and (**f**–**i**) seasonal average AOD over the PYRD from 2014–2017.

Figure 6a presents the spatial distribution of four-year average AOD over the PYRD from 2014 to 2017. The overall four-year average AOD over the PYRD was 0.514, with high values in the north-eastern and low in south-western. Shanghai and Jiangsu generally exhibited high AOD values, with a four-year average AOD of 0.626 and 0.622 respectively. Apart from the southeast and southwest, the four-year average AOD in most parts of Anhui was also large, ranging from 0.50 to 0.80. In contrast, Zhejiang was low in AOD values (the four-year average AOD was 0.395). Figure 6b–e indicate that spatial distribution in annual average AOD showed a similar pattern as the four-year average AOD. However, the average AOD over the PYRD showed a gradual decline. The high-AOD (>0.6) area decreased from 57.10% (of the total PYRD area) to 3.98% while the low-AOD (<0.3) area increased from 1.44% to 18.93% during the four years.

As illustrated in Figure 6f–i, AOD values for the four seasons were high in the north-eastern and low in the south-western. The average AOD in spring, summer, autumn, and winter over the PYRD were 0.544, 0.537, 0.467, and 0.500, respectively. Despite obvious seasonal variability, the high-AOD (>0.60) area was larger in spring (50.64% of the total PYRD area) and summer (46.7%). In autumn, the high-AOD (>0.60) area decreased obviously (8.43%) while the low-AOD (<0.3) area was largest in this season, accounting for 15.18% of the total PYRD area. In winter, most of the AOD values (60.3%) ranged from 0.5 to 0.7.

#### 4.2.2. Temporal Characteristics of AOD 

**Figure 7:**
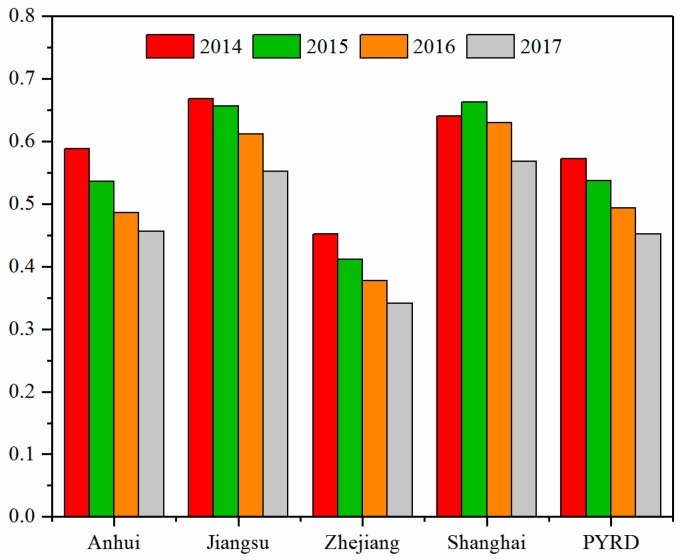
Annual average AOD over the PYRD from 2014 to 2017.

Figure 7 illustrates the changes in annual average AOD over the PYRD from 2014 to 2017. Decreasing trends were observed over Anhui (from 0.588 to 0.456), Jiangsu (from 0.669 to 0.552), Zhejiang (from 0.453 to 0.341), and the PYRD (from 0.573 to 0.452). Interestingly, the annual average AOD of Shanghai first rose from 0.641 in 2014 to 0.663 in 2015, and then declined to 0.568 in 2017.

**Figure 8:**
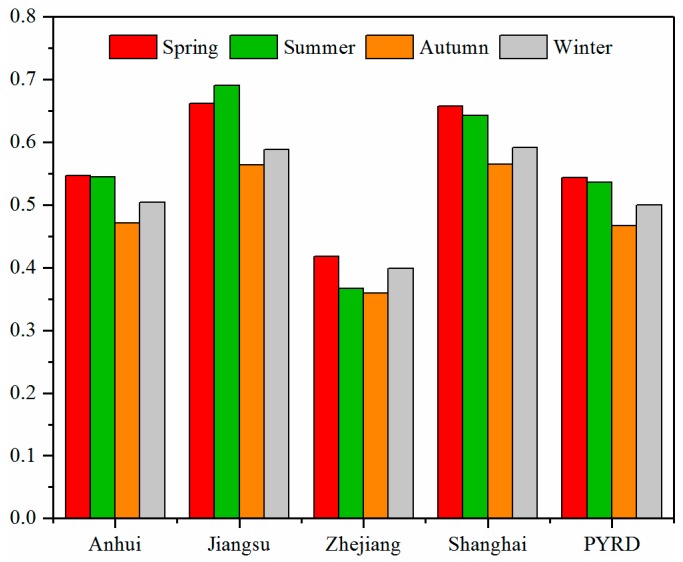
Seasonal average AOD over the PYRD and its four parts from 2014 to 2017.

Figure 8 demonstrates the seasonal variability in average AOD over the PYRD and its four parts in the four-year period. For Anhui, Zhejiang, Shanghai, and the PYRD, the seasonal average AOD were highest in spring (Anhui: 0.546, Zhejiang: 0.418, Shanghai: 0.658, PYRD: 0.544) while lowest in autumn (Anhui: 0.472, Zhejiang: 0.360, Shanghai: 0.565, PYRD: 0.467). In the case of Jiangsu, maximum seasonal average AOD was however observed in summer.

### 4.3. Contribution of Each Factor to AOD Distribution

**Figure 9:**
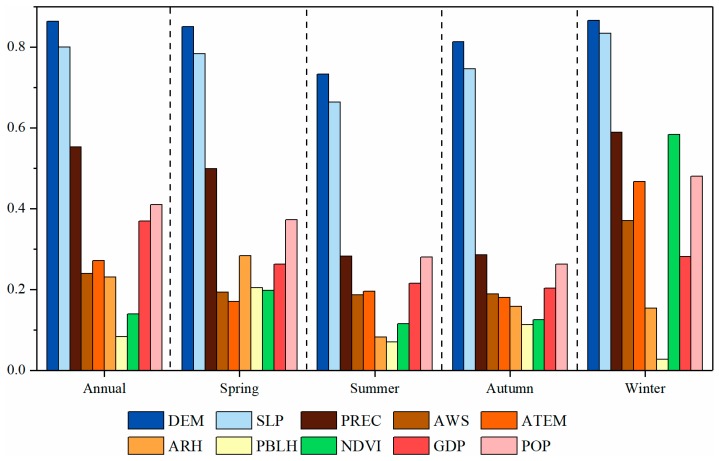
Seasonally and annually specific contribution of each factor to AOD over the PYRD. DEM: digital elevation model; SLP: slope; PREC: precipitation; AWS: average wind speed; ATEM: average temperature; ARH: average relative humidity; PBLH: planetary boundary layer height; NDVI: normalized difference vegetation index; GDP: gross domestic product; POP: population density.

The power of determinant values (q) were calculated by the geographical detector method and the impact directions were determined by multiple linear regression analysis. As shown in Figure 9, during the four-year period, the highest q value was found for DEM (0.863), with SLP a close second on 0.799, followed by PREC (0.553), POP (0.410), GDP (0.369), ATEM (0.271), AWS (0.239), ARH (0.231), NDVI (0.140) and PBLH (0.083).

In different seasons, the q values of DEM and SLP ranged from 0.733 to 0.866 and from 0.663 to 0.834, respectively, indicating they could explain AOD more than the other factors. The q values of PREC were 0.499, 0.282, 0.286, 0.588 in spring, summer, autumn, winter, respectively, which suggests that PREC was the main meteorological factor influencing AOD. AWS and ATEM also exhibited strong effects on AOD in winter, with the q value of 0.370 and 0.467, respectively. ARH and PBLH were found to show somewhat strong influences on AOD in spring (q value = 0.283 and 0.204 respectively). Notably, despite low in summer and autumn, the q value of NDVI reached a maximum of 0.583 in winter. In addition, GDP and POP exerted great influences on AOD distribution in all the seasons, with q values greater than 0.2.

The directions of regression coefficients for variables in multiple linear regression models (Table A5 in Appendix A) show the positive or negative correlation between AOD and the factors. DEM, PREC, NDVI and PBLH were found negatively linked to the AOD, while AWS, ARH, and POP were observed positively associated with the AOD. 

## 5. Discussion

### 5.1. AOD Gap-Filling

Previous studies mapped the spatiotemporal characteristics of AOD at coarse spatial resolutions, which makes it difficult to unravel regional-scale aerosol heterogeneity [16,43,66]. The latest released MODIS 3-km AOD can provide more fine-scale aerosol details over urban areas [27]. Due to the limitation of the Dark Target (DT) algorithm, there are however a large number of missing values in the daily AOD images of MODIS 3-km AOD [28]. In this study, we applied the method proposed by He and Huang [22] to merge AOD data at a regional-scale, and the accuracy and spatiotemporal coverage of merged AOD were compared. To our knowledge, this is the first attempt to merge 3-km DT and 10-km DB AOD with daily linear regression models at regional-scale. The linear regression models achieved high prediction accuracy (cross-validation R^2^ ranging from 0.82 to 0.88, Table A4). The performance of linear regression was steady across the years and among different AOD datasets (Table A4). Validation of merged AOD against AERONET AOD also indicate that when comparing with the original DT AOD, the merged AOD (both for Terra and Aqua) outperformed in RMSE and the percentage of AOD retrievals within EE. The correlation coefficients (R), though a slight decrease after merging, were higher in our study (>0.91 for both Terra and Aqua) than those reported by Qin et al. (R = 0.71) [40] and Wang et al. (R = 0.84) [66], enough to allow the investigation of spatiotemporal variations of aerosols. 

Meanwhile, the coverage of the merged AOD was improved in this study. The average spatial coverage increased from 13.7% (the original Terra DT AOD) and 11.45% (the original Aqua DT AOD) to 26.52% (Figure A1). In a previous study, Xu et al. [37] merged MISR, SeaWiFS, MODIS DT, MODIS DB and MODIS SRAP AOD datasets using the maximum likelihood estimate method over Asia for the year 2007, with the average spatial coverage of AOD data increasing to 50% while the figures of the operational AOD datasets only ranged from 5% to 20%. Compared with the study of Xu et al., the coverage improvement of our study is slightly lower, probably due to the low availability of the original AOD data [85]. The linear regression merging approach could only estimate the AOD pixels at where at least one of the four AOD datasets has valid retrievals. However, because of the cloud contamination and retrieval errors [32], the original AOD datasets have large numbers of data gaps in the PYRD. The mean daily spatial coverage of the original Terra DT, Aqua DT, Terra DB and Aqua DB data were 13.7%, 11.45%, 19.98% and 16.91% respectively (Figure A1). Insufficient retrievals lead to limited improvement in AOD coverage. Moreover, the more datasets utilized in the study of Xu et al. broadened the coverage of AOD data to a greater degree. Additionally, the temporal coverage for most of the PYRD increased to 20%-40% in our study (Figure 5), which were similar to the result of He and Huang [22]. Though limited, the linear regression merging approach improved the spatial and temporal coverage to a certain degree, which could provide more information about AOD for the subsequent analyses (i.e., spatiotemporal variations and influencing factors analysis of AOD). The results of our study also proved that this method is not only suitable for merging AOD at national-scale but regional-scale.

### 5.2. The Impacts of Factors on the Spatial Variations of AOD

Topography was confirmed to be closely and negatively related to AOD, with quite high q values for DEM and SLP (Figure 9, Table A5). Over the PYRD, high AOD values were observed in plain and tableland areas (Figure 6a) such as Jiangsu, Shanghai, and North, and Central Anhui, while low AOD values were primarily concentrated in hilly areas like Zhejiang, Southwest and Southeast Anhui. A previous study has found that both the MODIS C6.1 DT and DB AOD retrievals show small biases in low-elevation areas (height < 800 m) while the DT AOD retrievals show increasing positive biases as the elevation increases in high-elevation areas (height > 800 m) [26]. Since most parts of the PYRD (approximately 97.47%) are at elevations below 800 meters, it was assumed that biases caused by elevation are small. The close and negative association between DEM, SLP and AOD may be explained from three aspects: firstly, low-elevation and flat areas are more influenced by human activity such as industry and construction, and thus emitted more air pollutants [34,50]; secondly, high mountains in high-elevation areas can prevent the horizontal dispersion of air pollutants [19,96]; and lastly, for the mid-latitude areas, precipitation usually increases with elevation [97], while precipitation is capable of bringing down aerosols [46]. Previous studies also showed the aerosol distribution is strongly affected by topography conditions [19,41,42,43].

Compared to other factors, socioeconomic factors (i.e., population density and GDP) were identified as greater contributors to AOD (Figure 9, Table A5), explaining 41% and 36.9% of the spatial variability in AOD, respectively. This finding agrees with previous studies of AOD-GDP association in Guangdong [50], Huaihai economic region [45] and mainland China [40] and AOD-population density association in mainland China [40]. It is due to the fact that a dense population causes high anthropogenic aerosol particles emissions and that high GDP requires heavy energy consumption [40,65], thereby leading to an increase in AOD. For example, Jiangsu and Shanghai were areas with large populations and high GDP in China, where an enormous amount of fuel combustion, industrial emission, and transportation and construction sources have always caused large AOD values [49,65]. But despite high population density and GDP, Zhejiang had lower AOD values than the other areas. We assume that the possible reason is that the impact of terrain was stronger than socioeconomic factors.

The influence of local meteorological factors on the spatial pattern of AOD varied in different periods. From the result of geographical detector method and multiple linear regression analysis (Figure 9, Table A5), precipitation had a prominent negative impact on the AOD during the four-year period and in each season, which is consistent with multiple previous studies [19,44,45,46]. It is because precipitation can lower aerosol concentration by washing away aerosols [45]. Additionally, precipitation tends to increase soil moisture, making the dust more difficult to rise into the atmosphere [19]. The influence of wind speed on AOD is complex because it may either disperse aerosols or bring in fresh aerosols [47,48]. Wind speed was observed to make an important positive contribution to AOD, particularly in winter. The prevailing north-west wind in winter can bring in highly polluted airborne particles from North China to the PYRD [66]. The planetary boundary layer height exhibited an obvious negative impact on AOD in spring. This is due to the fact that relative high planetary boundary layer in this season can lead to strong dilution and diffusion of aerosol particles [53]. In addition, relative humidity and temperature also have strong impacts on AOD in spring and in winter, respectively. It was well documented that the higher relative humidity could result in a larger volume of fine particles because of the hygroscopic growth of aerosol particles [19,51]. Regarding temperature, some studies have confirmed that high temperature can promote the photochemical reaction, thus increasing aerosol concentrations in the atmosphere [49,50,51]. However, on the other hand, the occurrence of inversion phenomenon in winter may hinder the diffusion and dispersion of aerosol particles, causing accumulation of aerosols over the region [48,98]. In the present study, since the temperature was not included in the models, thus its impact directions were not detected. Previous studies have reported that AOD is strongly and negatively related to the NDVI in Guangdong and Yangtze River Basin [44,50], because denser vegetation can lower AOD values by absorbing and depositing aerosol particles, especially in the dusty environment [19]. In some cases, however, vegetation can also increase AOD, for example, through burning straw in rural areas [19,44,52,99]. In our study, NDVI contributed to the AOD negatively in winter more than in the other seasons (Figure 9, Table A5). A possible explanation is that owing to the sparse vegetation in winter, large amounts of dust aerosols were emitted into the air by wind erosion and this remarkably increased the aerosols in the atmosphere. In contrast, thick vegetation in spring, summer, and autumn mitigated the determinate power of NDVI for the spatial variability of AOD in these seasons.

### 5.3. The Effect of Environmental Policy on the Temporal Variability of AOD

The annual average AOD of the PYRD showed a decreasing trend from 2014 to 2017, in agreement with the trends observed in the Huaihai Economic Region [45] and East China [49]. It has been widely acknowledged that precipitation, temperature, and wind speed can impact the concentration of aerosols [16,19,51]. However, no prominent annual variation on precipitation, wind speed, or temperature over the PYRD was observed during the 2014–2017 period. Hence, the decline of annual average AOD might not be attributed to temporal change in meteorological factors. On the other hand, ground-level particles have presented a downward trend in recent years, which might be a key reason for the reduction in annual average AOD [42]. Since 2013, a variety of environmental measures have been implemented to lower PM emissions in the PYRD by the central and local governments, for instance, improving combustion technologies and vehicle emission standards, adjusting the energy structure, and utilizing clear energy [42,66,98].

### 5.4. Limitations

There are some limitations in this study. Firstly, though the linear regression-based merging approach can improve the spatiotemporal coverage of MODIS AOD, large data gaps remain in some daily images. Thus, the other gap-filling methods, such as spatiotemporal kriging [34] and multiple imputation [32] should be adopted to further fill AOD based on MODIS AOD merging. Secondly, we only focused on the impacts of factors on the spatial pattern of AOD, without considering the causes for seasonal variations of AOD. Lastly, although anthropogenic emissions are prominent sources of atmospheric aerosols, we only considered two socioeconomic factors (GDP and population density) due to the lack of data. More factors should be selected to represent the impact of human activity.

## 6. Conclusions

In this study, we merged four MODIS AOD datasets from 2014 to 2017 with an assessment of the accuracy and spatiotemporal coverage of the merged AOD and investigated its spatial pattern and temporal variations over the Pan Yangtze River Delta (PYRD). In addition, the contributions of topography, meteorology, vegetation, and socioeconomic factors to AOD distribution were identified through the geographical detector method and multiple linear regression analysis. The key findings and main conclusions are as follows:

- The merged AOD are better than the original Terra/Aqua DT AOD, with the average spatial coverage increased by 94% and 132% respectively.
- The AOD over the PYRD were high in the northeast and low in the southwest and decreased from 2014 to 2017. Seasonal average AOD were relatively higher in spring and summer than in autumn and winter.
- Topographical factors contributed most to AOD, followed by precipitation and population density, while NDVI showed a relatively week impact on AOD.

Our study highlights how AOD varies over time and in space and therefore, has the potential to contribute to the formulation of environmental policy to protect atmospheric quality over large economically prosperous regions like the PYRD.

## Figures and Tables

**Table 1:** MODIS AOD data products used in this study.

AOD Data Products Types	Scientific Data Set (SDS)	Contents	Temporal Range	Use
Terra/Aqua 3-km DT AOD	Optical_Depth_Land_And_Ocean	DT AOD (QA = 3)	2005.1.1–2013.12.31	Calibration
			2014.1.1–2017.12.31	Spatiotemporal characteristics and influencing factors analysis
Terra/Aqua 10-km DB AOD	Deep_Blue_Aerosol_Optical_Depth_550_Land_Best_Estimate	DB AOD (QA ≥ 2)	2005.1.1–2013.12.31	Calibration
			2014.1.1–2017.12.31	Spatiotemporal characteristics and influencing factors analysis

MODIS = Moderate Resolution Imaging Spectrometers. DT AOD = Dark Target aerosol optical depth. DB AOD = Deep Blue aerosol optical depth.

**Table 2:** Locations of the Aerosol Robotic Network (AERONET) sites within the Pan Yangtze River Delta (PYRD) (see Figure 1) and the periods of their available data.

Number	Site Name	Longitude (°N)	Latitude (°E)	Elevation (m)	Period of Available Data
1	XuZhou-CUMT	117.1417	34.2167	59.7	2013–2017
2	Shouxian	116.7820	32.5584	22.7	2008
3	Hefei	117.1622	31.9047	36	2005–2008, 2016
4	NUIST	118.7172	32.2065	62	2007–2010
5	SONET_Nanjing	118.9570	32.1150	52	2016
6	Taihu	120.2153	31.4210	20	2005–2017
7	SONET_Shanghai	121.4810	31.2840	24	2016
8	Shanghi_Minhang	121.3973	31.1305	49	2008–2009
9	Shanghi_Met	121.5485	31.2214	5	2007
10	Hangzhou_City	120.1569	30.2896	30	2008–2009
11	Hangzhou-ZFU	119.7274	30.2572	42	2007–2009
12	LA-TM	119.4400	30.3240	439	2007–2009
13	Qiandaohu	119.0526	29.5557	133	2007–2008
14	Ningbo	121.5469	29.8599	37	2007–2008
15	SONET_Zhoushan	122.1880	29.9940	29	2016

## Abbreviations

AOD: Aerosol optical depth; DEM: Digital Elevation Model; DT: Dark Target; SLP: Slope; DB: Deep Blue; PREC: Precipitation; MODIS: Moderate Resolution Imaging Spectrometers; ATEM: Average temperature; AERONET: Aerosol Robotic Network; AWS: Average wind speed; CV: Cross-validation; ARH: Average relative humidity; RMSE: Root mean squared error; PBLH: Planetary boundary layer height; RPE: Relative prediction error; NDVI: Normalized difference vegetation index; R^2^: Coefficient of determination; GDP: Gross domestic product; R: Correlation efficient; POP: Population density; VIF: Variance Inflation Factor.

## Appendix A

### Validation of the Resampled 3-km DB AOD Data

The 3-km DB AOD data from 2014 to 2017, resampled by nearest neighbor, bilinear interpolation and cubic convolution methods respectively, were validated against AERONET AOD values.

**Table A1:** Validation summary of the resampled 3-km DB AOD data.

AOD	Nearest Neighbor		Bilinear Interpolation		Cubic Convolution	
	$R^{2}$	RMSE	$R^{2}$	RMSE	$R^{2}$	RMSE
Terra DB AOD	0.78	0.16	0.77	0.17	0.77	0.17
Aqua DB AOD	0.82	0.17	0.81	0.18	0.78	0.20

### The Calibration of AOD

The linear regression relationships between the four MODIS AOD datasets and AERONET AOD data were established using the following equation:

(6) $$AOD_{Aeronet}=a+b\times AOD_{MODIS},$$

where $AOD_{Aeronet}$ refers to AERONET AOD data, $AOD_{MODIS}$ refers to Terra DT, Aqua DT, Terra DB or Aqua DB AOD data; a, b, $R^{2}$ refer to intercept, slope, and R square of the linear regression respectively.

**Table A2:** Linear regression models for the four MODIS AOD datasets calibration.

Seasons	Terra DT AOD			Aqua DT AOD			Terra DB AOD			Aqua DB AOD		
	a	b	$R^{2}$	a	b	$R^{2}$	a	b	$R^{2}$	a	b	$R^{2}$
Spring	0.01	0.75	0.82	0.03	0.77	0.78	0.19	0.85	0.83	0.17	0.90	0.77
Summer	−0.12	1.0	0.89	−0.08	0.93	0.80	0.17	0.95	0.89	0.14	0.95	0.86
Autumn	0.03	0.86	0.84	0.12	0.71	0.84	0.17	0.76	0.84	0.21	0.63	0.76
Winter	0.05	0.86	0.76	0.11	0.76	0.82	0.18	0.67	0.87	0.16	0.77	0.81

**Table A3:** Pearson correlations between AERONET AOD values at times when two satellites overpass.

Year	*N*	R
2014	115	0.8462
2015	129	0.8734
2016	116	0.8324
2017	132	0.8267
2014–2017	492	0.8477

*N*: the number of samples; R: Pearson correlation coefficients between AERONET AOD values at times when two satellites overpass.

**Table A4:** Predictive performance of the linear regression models for missing AOD data and standard deviation of the predicted AOD values (10-fold cross-validation).

Model	Year	RMSE	RPE (%)	$R^{2}$	σ
Predict Terra DT AOD with Aqua DT AOD	2014	0.14	28.8	0.83	0.0054
	2015	0.12	27.5	0.85	0.0017
	2016	0.12	29.6	0.83	0.0051
	2017	0.10	28.7	0.82	0.0064
	2014−2017	0.12	28.8	0.83	0.0110
Predict Aqua DT AOD with Terra DT AOD	2014	0.13	26.3	0.82	0.0028
	2015	0.11	24.8	0.85	0.0019
	2016	0.11	27.3	0.83	0.0032
	2017	0.10	24.7	0.83	0.0050
	2014−2017	0.11	25.9	0.83	0.0061
Predict Terra DB AOD with Aqua DB AOD	2014	0.11	23.5	0.87	0.0024
	2015	0.10	21.0	0.85	0.0015
	2016	0.10	21.2	0.86	0.0018
	2017	0.09	21.6	0.83	0.0013
	2014−2017	0.10	22.1	0.86	0.0028
Predict Aqua DB AOD with Terra DB AOD	2014	0.12	23.6	0.88	0.0010
	2015	0.10	20.8	0.85	0.0003
	2016	0.10	21.5	0.85	0.0034
	2017	0.09	21.2	0.84	0.0002
	2014−2017	0.10	22.0	0.86	0.0011
Predict Terra DT AOD with Terra DB AOD	2014	0.12	23.7	0.86	0.0021
	2015	0.12	23.0	0.85	0.0014
	2016	0.11	24.8	0.85	0.0030
	2017	0.10	25.4	0.84	0.0022
	2014−2017	0.12	24.2	0.86	0.0045
Predict Aqua DT AOD with Aqua DB AOD	2014	0.11	21.9	0.86	0.0044
	2015	0.11	21.2	0.85	0.0032
	2016	0.11	23.9	0.84	0.0023
	2017	0.10	22.5	0.83	0.0012
	2014−2017	0.11	22.4	0.85	0.0037

### Multiple Linear Regression Models

To avoid the collinearity issue, Pearson correlation analyses among influencing factors were conducted for each season and the four-year period. If two factors were closely correlated (Pearson’s r > 0.6) with each other, only one of the two was selected to develop the final model while the other was removed. For example, the results of Pearson correlation analyses show that some factors (i.e., DEM vs. SLP, PREC vs. ARH, AWS vs. ATEM, and POP vs. GDP) were closely correlated in spring. Meanwhile, the results of multiple linear regression models show that DEM, PREC, AWS and POP have better performance than the others. Thus, we selected DEM, PREC, AWS, POP, PBLH, and NDVI in the spring model (Table A5). The same method was used to build models for the other seasons and the whole period (Table A5).

**Table A5:** Multiple linear regression analysis of season (annual) mean AOD and standardized impact factors.

Model	Regression Function	$R^{2}$	Adjusted $R^{2}$	Max VIF (Variable)
Annual	AOD = 2.492 × 10^−15^ 0.566 × DEM − 0.307 × PREC + 0.098 × AWS − 0.025 × PBLH − 0.076 × NDVI + 0.210 × POP	0.792	0.792	2.365 (DEM)
Spring	AOD = −9.663 × 10^−16^ − 0.500 × DEM − 0.265 × PREC + 0.103 × AWS − 0.127 × PBLH − 0.118 × NDVI + 0.164 × POP	0.806	0.806	2.817 (DEM)
Summer	AOD = 3.121 × 10−15 − 0.616 × DEM − 0.202 × PREC + 0.173 × AWS + 0.099 × ARH - 0.032 × PBLH − 0.102 × NDVI + 0.247 × POP	0.677	0.677	2.061 (ARH)
Autumn	AOD = 1.445 × 10−15 − 0.639 × DEM − 0.348 × PREC + 0.188 × AWS + 0.081 × ARH − 0.068 × PBLH − 0.072 × NDVI + 0.324 × POP	0.824	0.823	2.411 (PREC)
Winter	AOD = 4.822 × 10−16 − 0.523 × DEM − 0.250 × PREC + 0.110 × AWS − 0.090 × PBLH − 0.234 × NDVI + 0.228 × POP	0.833	0.833	2.832 (DEM)

VIF: Variance Inflation Factor. VIF of each independent variable less than 3 indicates that there is no collinearity in the regression model.

## References

[1] Hallquist M., Wenger J.C., Baltensperger U., Rudich Y., Simpson D., Claeys M., Dommen J., Donahue N.M., George C., Goldstein A.H. (2009). The formation, properties and impact of secondary organic aerosol: Current and emerging issues. Atmos. Chem. Phys..

[2] Viana M., Pey J., Querol X., Alastuey A., de Leeuw F., Lükewille A. (2014). Natural sources of atmospheric aerosols influencing air quality across Europe. Sci. Total Environ..

[3] Li L., Solana C., Canters F., Chan J., Kervyn M. (2015). Impact of environmental factors on the spectral characteristics of lava surfaces: Field spectrometry of basaltic lava flows on Tenerife, Canary Islands, Spain. Remote Sens..

[4] Calvo A.I., Alves C., Castro A., Pont V., Vicente A.M., Fraile R. (2013). Research on aerosol sources and chemical composition: Past, current and emerging issues. Atmos. Res..

[5] Andreae M.O., Rosenfeld D. (2008). Aerosol-cloud-precipitation interactions. Part 1. The nature and sources of cloud-active aerosols. Earth-Sci. Rev..

[6] Kaufman Y.J., Tanré D., Boucher O. (2002). A satellite view of aerosols in the climate system. Nature.

[7] Obregón M.A., Serrano A., Cancillo M.L., Cachorro V.E., Toledano C. (2015). Aerosol radiometric properties at Western Spain (Cáceres station). Int. J. Climatol..

[8] Cheung H.C., Wang T., Baumann K., Guo H. (2005). Influence of regional pollution outflow on the concentrations of fine particulate matter and visibility in the coastal area of Southern China. Atmos. Environ..

[9] Park R.J., Jacob D.J., Kumar N., Yantosca R.M. (2006). Regional visibility statistics in the United States: Natural and transboundary pollution influences, and implications for the Regional Haze Rule. Atmos. Environ..

[10] Bäumer D., Vogel B., Versick S., Rinke R., Möhler O., Schnaiter M. (2008). Relationship of visibility, aerosol optical thickness and aerosol size distribution in an ageing air mass over South-West Germany. Atmos. Environ..

[11] Yu L.E., Shulman M.L., Kopperud R., Hildemann L.M. (2005). Characterization of organic compounds collected during Southeastern aerosol and visibility study: Water-soluble organic species. Environ. Sci. Technol..

[12] Harrison R.M., Yin J. (2000). Particulate matter in the atmosphere: Which particle properties are important for its effects on health?. Sci. Total Environ..

[13] Pope C.A., Dockery D.W. (2006). Health effects of fine particulate air pollution: Lines that connect. J. Air Waste Manag. Assoc..

[14] Dinoi A., Perrone M.R., Burlizzi P. (2010). Application of MODIS products for air quality studies over Southeastern Italy. Remote Sens..

[15] Seo S., Kim J., Lee H., Jeong U., Kim W., Holben B.N., Kim S.W., Song C.H., Lim J.H. (2015). Estimation of PM_10_ concentrations over Seoul using multiple empirical models with AERONET and MODIS data collected during the DRAGON-Asia campaign. Atmos. Chem. Phys..

[16] Liu X., Chen Q., Che H., Zhang R., Gui K., Zhang H., Zhao T. (2016). Spatial distribution and temporal variation of aerosol optical depth in the Sichuan basin, China, the recent ten years. Atmos. Environ..

[17] Estellés V., Campanelli M., Utrillas M.P., Expósito F., Martínez-Lozano J.A. (2012). Comparison of AERONET and SKYRAD4.2 inversion products retrieved from a Cimel CE318 sunphotometer. Atmos. Meas. Tech..

[18] Cesnulyte V., Lindfors A.V., Pitkänen M.R.A., Lehtinen K.E.J., Morcrette J.-J., Arola A. (2014). Comparing ECMWF AOD with AERONET observations at visible and UV wavelengths. Atmos. Chem. Phys..

[19] Aklesso M., Kumar K.R., Bu L., Boiyo R. (2018). Analysis of spatial-temporal heterogeneity in remotely sensed aerosol properties observed during 2005–2015 over three countries along the Gulf of Guinea Coast in Southern West Africa. Atmos. Environ..

[20] Nichol J., Bilal M. (2016). Validation of MODIS 3 km resolution aerosol optical depth retrievals over Asia. Remote Sens..

[21] Ma Z., Hu X., Huang L., Bi J., Liu Y. (2014). Estimating ground-level PM_2.5_ in China using satellite remote sensing. Environ. Sci. Technol..

[22] He Q., Huang B. (2018). Satellite-based mapping of daily high-resolution ground PM_2.5_ in China via space-time regression modeling. Remote Sens. Environ..

[23] Tao M., Chen L., Wang Z., Tao J., Che H., Wang X., Wang Y. (2015). Comparison and evaluation of the MODIS Collection 6 aerosol data in China. J. Geophys. Res. Atmos..

[24] Fan A., Chen W., Liang L., Sun W., Lin Y., Che H., Zhao X. (2017). Evaluation and comparison of long-term MODIS C5.1 and C6 products against AERONET observations over China. Remote Sens..

[25] Bilal M., Qiu Z., Campbell J., Spak S., Shen X., Nazeer M. (2018). A new MODIS C6 Dark Target and Deep Blue merged aerosol product on a 3 km spatial grid. Remote Sens..

[26] Wei J., Li Z., Peng Y., Sun L. (2019). MODIS Collection 6.1 aerosol optical depth products over land and ocean: Validation and comparison. Atmos. Environ..

[27] Remer L.A., Mattoo S., Levy R.C., Munchak L.A. (2013). MODIS 3 km aerosol product: Algorithm and global perspective. Atmos. Meas. Tech..

[28] He Q., Zhang M., Huang B., Tong X. (2017). MODIS 3 km and 10 km aerosol optical depth for China: Evaluation and comparison. Atmos. Environ..

[29] Hsu N.C., Jeong M.J., Bettenhausen C., Sayer A.M., Hansell R., Seftor C.S., Huang J., Tsay S.C. (2013). Enhanced Deep Blue aerosol retrieval algorithm: The second generation. J. Geophys. Res. Atmos..

[30] Sayer A.M., Munchak L.A., Hsu N.C., Levy R.C., Bettenhausen C., Jeong M.-J. (2014). MODIS Collection 6 aerosol products: Comparison between Aqua’s e-Deep Blue, Dark Target, and “merged” data sets, and usage recommendations. J. Geophys. Res. Atmos..

[31] Lee H.J., Liu Y., Coull B.A., Schwartz J., Koutrakis P. (2011). A novel calibration approach of MODIS AOD data to predict PM_2.5_ concentrations. Atmos. Chem. Phys..

[32] Xiao Q., Wang Y., Chang H.H., Meng X., Geng G., Lyapustin A., Liu Y. (2017). Full-coverage high-resolution daily PM_2.5_ estimation using MAIAC AOD in the Yangtze River Delta of China. Remote Sens. Environ..

[33] Ruiz-Arias J.A., Dudhia J., Lara-Fanego V., Pozo-Vázquez D. (2013). A geostatistical approach for producing daily Level-3 MODIS aerosol optical depth analyses. Atmos. Environ..

[34] Yang J., Hu M. (2018). Filling the missing data gaps of daily MODIS AOD using spatiotemporal interpolation. Sci. Total Environ..

[35] Lv B., Hu Y., Chang H.H., Russell A.G., Cai J., Xu B., Bai Y. (2017). Daily estimation of ground-level PM_2.5_ concentrations at 4 km resolution over Beijing-Tianjin-Hebei by fusing MODIS AOD and ground observations. Sci. Total Environ..

[36] Nirala M. (2008). Technical Note: Multi-sensor data fusion of aerosol optical thickness. Int. J. Remote Sens..

[37] Xu H., Guang J., Xue Y., de Leeuw G., Che Y.H., Guo J., He X.W., Wang T.K. (2015). A consistent aerosol optical depth (AOD) dataset over mainland China by integration of several AOD products. Atmos. Environ..

[38] Ma Z., Hu X., Sayer A.M., Levy R., Zhang Q., Xue Y., Tong S., Bi J., Huang L., Liu Y. (2016). Satellite-based spatiotemporal trends in PM_2.5_ concentrations: China, 2004–2013. Environ. Health Perspect..

[39] Zheng Y., Zhang Q., Liu Y., Geng G., He K. (2016). Estimating ground-level PM_2.5_ concentrations over three megalopolises in China using satellite-derived aerosol optical depth measurements. Atmos. Environ..

[40] Qin W., Liu Y., Wang L., Lin A., Xia X., Che H., Bilal M., Zhang M. (2018). Characteristic and driving factors of aerosol optical depth over Mainland China during 1980–2017. Remote Sens..

[41] Shi H., He Q., Zhang W. (2018). Spatial factor analysis for aerosol optical depth in metropolises in China with regard to spatial heterogeneity. Atmosphere.

[42] He Q., Gu Y., Zhang M. (2019). Spatiotemporal patterns of aerosol optical depth throughout China from 2003 to 2016. Sci. Total Environ..

[43] Rezaei M., Farajzadeh M., Mielonen T., Ghavidel Y. (2019). Analysis of spatio-temporal dust aerosol frequency over Iran based on satellite data. Atmos. Pollut. Res..

[44] Guo Y., Hong S., Feng N., Zhuang Y., Zhang L. (2012). Spatial distributions and temporal variations of atmospheric aerosols and the affecting factors: A case study for a region in Central China. Int. J. Remote Sens..

[45] Shen Y., Zhang L., Fang X., Zhao Z., Li X., Wang J., Chai Q. (2018). Long-term analysis of aerosol optical depth over the Huaihai Economic Region (HER): Possible causes and implications. Atmosphere.

[46] Papadimas C.D., Hatzianastassiou N., Mihalopoulos N., Querol X., Vardavas I. (2008). Spatial and temporal variability in aerosol properties over the Mediterranean basin based on 6-year (2000–2006) MODIS data. J. Geophys. Res..

[47] Gunaseelan I., Bhaskar B.V., Muthuchelian K. (2014). The effect of aerosol optical depth on rainfall with reference to meteorology over metro cities in India. Environ. Sci. Pollut. Res..

[48] Soni M., Payra S., Verma S. (2018). Particulate matter estimation over a semi arid region Jaipur, India using satellite AOD and meteorological parameters. Atmos. Pollut. Res..

[49] Wang P., Ning S., Dai J., Sun J., Lv M., Song Q., Dai X., Zhao J., Yu D. (2017). Trends and variability in aerosol optical depth over North China from MODIS C6 aerosol products during 2001–2016. Atmosphere.

[50] Li L., Wang Y. (2015). What drives the aerosol distribution in Guangdong—The most developed province in Southern China?. Sci. Rep..

[51] Che H., Zhang X., Alfraro S., Chatenet B., Gomes L., Zhao J. (2009). Aerosol optical properties and its radiative forcing over Yulin, China in 2001 and 2002. Adv. Atmos. Sci..

[52] Deng X., Shi C., Wu B., Chen Z., Nie S., He D., Zhang H. (2012). Analysis of aerosol characteristics and their relationships with meteorological parameters over Anhui province in China. Atmos. Res..

[53] Li X., Song H., Zhai S., Lu S., Kong Y., Xia H., Zhao H. (2019). Particulate matter pollution in Chinese cities: Areal-temporal variations and their relationships with meteorological conditions (2015–2017). Environ. Pollut..

[54] Lanzaco B.L., Olcese L.E., Palancar G.G., Toselli B.M. (2016). A method to improve MODIS AOD values: Application to South America. Aerosol Air Qual. Res..

[55] Comber A., Chi K., Huy M.Q., Nguyen Q., Lu B., Phe H.H., Harris P. (2018). Distance metric choice can both reduce and induce collinearity in geographically weighted regression. Environ. Plan. B Urban Anal. City Sci..

[56] Luo L., Mei K., Qu L., Zhang C., Chen H., Wang S., Di D., Huang H., Wang Z., Xia F. (2019). Assessment of the geographical detector method for investigating heavy metal source apportionment in an urban watershed of Eastern China. Sci. Total Environ..

[57] Wang J., Li X., Christakos G., Liao Y., Zhang T., Gu X., Zheng X. (2010). Geographical detectors-based health risk assessment and its application in the neural tube defects study of the Heshun Region, China. Int. J. Geogr. Inf. Sci..

[58] Cao Z., Liu T., Li X., Wang J., Lin H., Chen L., Wu Z., Ma W. (2017). Individual and interactive effects of socio-ecological factors on dengue fever at fine spatial scale: A geographical detector-based analysis. Int. J. Environ. Res. Public Health.

[59] Zhou C., Chen J., Wang S. (2018). Examining the effects of socioeconomic development on fine particulate matter (PM_2.5_) in China’s cities using spatial regression and the geographical detector technique. Sci. Total Environ..

[60] Ding Y., Zhang M., Qian X., Li C., Chen S., Wang W. (2019). Using the geographical detector technique to explore the impact of socioeconomic factors on PM_2.5_ concentrations in China. J. Clean. Prod..

[61] Song X., Hao Y., Zhang C., Peng J., Zhu X. (2016). Vehicular emission trends in the Pan-Yangtze River Delta in China between 1999 and 2013. J. Clean. Prod..

[62] Wu Y., Zhu X., Gao W., Qian F. (2018). The spatial characteristics of coupling relationship between urbanization and eco-environment in the Pan Yangtze River Delta. Energy Procedia.

[63] Cui Y., Li L., Chen L., Zhang Y., Cheng L., Zhou X., Yang X. (2018). Land-use carbon emissions estimation for the Yangtze River Delta urban agglomeration using 1994–2016 Landsat image data. Remote Sens..

[64] Ma Z., Liu Y., Zhao Q., Liu M., Zhou Y., Bi J. (2016). Satellite-derived high resolution PM_2.5_ concentrations in Yangtze River Delta Region of China using improved linear mixed effects model. Atmos. Environ..

[65] Yun G., He Y., Jiang Y., Dou P., Dai S. (2019). PM_2.5_ spatiotemporal evolution and drivers in the Yangtze River Delta between 2005 and 2015. Atmosphere.

[66] Wang X., Guo Z., Wang Y., Chen Y., Zheng X., Xu X. (2018). Monitoring temporal–spatial variations of AOD over the Yangtze River Delta, China. Stoch. Environ. Res. Risk Assess..

[67] Xia H., Wang H., Ji G. (2019). Regional inequality and influencing factors of primary PM emissions in the Yangtze River Delta, China. Sustainability.

[68] Xu Y., Xu Y., Wang Y., Wu L., Li G., Song S. (2017). Spatial and temporal trends of reference crop evapotranspiration and its influential variables in Yangtze River Delta, eastern China. Theor. Appl. Climatol..

[69] National Bureau of Statistics, Ministry of Environmental Protection of the People’s Republic of China (2015). China Statistical Yearbook on Environment 2015.

[70] Ministry of Environmental Protection, General Administration of Quality Supervision, Inspection and Quarantine of the People’s Republic of China (2012). Ambient Air Quality Standards (GB3095-2012).

[71] Jung C., Hwang B., Chen W. (2018). Incorporating long-term satellite-based aerosol optical depth, localized land use data, and meteorological variables to estimate ground-level PM_2.5_ concentrations in Taiwan from 2005 to 2015. Environ. Pollut..

[72] Level 1 and Atmosphere Archive and Distribution System. https://ladsweb.nascom.nasa.gov/.

[73] Levy R.C., Mattoo S., Munchak L.A., Remer L.A., Sayer A.M., Patadia F., Hsu N.C. (2013). The Collection 6 MODIS aerosol products over land and ocean. Atmos. Meas. Tech..

[74] Aerosol Robotic Network. https://aeronet.gsfc.nasa.gov/.

[75] Holben B.N., Eck T.F., Slutsker I., Tanré D., Buis J.P., Setzer A., Vermote E., Reagan J.A., Kaufman Y.J., Nakajima T. (1998). AERONET—A federated instrument network and data archive for aerosol characterization. Remote Sens. Environ..

[76] Holben B.N., Tanré D., Smirnov A., Eck T.F., Slutsker I., Abuhassan N., Newcomb W.W., Schafer J.S., Chatenet B., Lavenu F. (2001). An emerging ground-based aerosol climatology: Aerosol optical depth from AERONET. J. Geophys. Res. Atmos..

[77] Ichoku C. (2002). A spatio-temporal approach for global validation and analysis of MODIS aerosol products. Geophys. Res. Lett..

[78] Prados A.I., Kondragunta S., Ciren P., Knapp K.R. (2007). GOES Aerosol/Smoke Product (GASP) over North America: Comparisons to AERONET and MODIS observations. J. Geophys. Res..

[79] Ma Y., Li Z., Li Z., Xie Y., Fu Q., Li D., Zhang Y., Xu H., Li K. (2016). Validation of MODIS aerosol optical depth retrieval over mountains in Central China based on a sun-sky radiometer site of SONET. Remote Sens..

[80] Chu D.A., Kaufman Y.J., Ichoku C. (2002). Validation of MODIS aerosol optical depth retrieval over land. Geophys. Res. Lett..

[81] Consultative Group for International Agriculture Research Consortium for Spatial Information. http://srtm.csi.cgiar.org/.

[82] China Meteorological Data Service Center. http://data.cma.cn/en.

[83] European Center for Medium-Range Weather Forecasts. http://www.ecmwf.int/.

[84] Data Center for Resources and Environmental Sciences, Chinese Academy of Sciences. http://www.resdc.cn/.

[85] Tang Q., Bo Y., Zhu Y. (2016). Spatiotemporal fusion of multiple-satellite aerosol optical depth (AOD) products using Bayesian maximum entropy method. J. Geophys. Res. Atmos..

[86] Jinnagara Puttaswamy S., Nguyen H.M., Braverman A., Hu X., Liu Y. (2014). Statistical data fusion of multi-sensor AOD over the Continental United States. Geocarto Int..

[87] Zhang R., Di B., Luo Y., Deng X., Grieneisen M.L., Wang Z., Yao G., Zhan Y. (2018). A nonparametric approach to filling gaps in satellite-retrieved aerosol optical depth for estimating ambient PM_2.5_ levels. Environ. Pollut..

[88] Wei J., Li Z., Peng Y., Sun L., Yan X. (2019). A regionally robust high-spatial-resolution aerosol retrieval algorithm for MODIS images over Eastern China. IEEE Trans. Geosci. Remote Sens..

[89] Meng C., Cheng T., Gu X., Shi S., Wang W., Wu Y., Bao F. (2019). Contribution of meteorological factors to particulate pollution during winters in Beijing. Sci. Total Environ..

[90] Meng X., Fu Q., Ma Z., Chen L., Zou B., Zhang Y., Xue W., Wang J., Wang D., Kan H. (2016). Estimating ground-level PM_10_ in a Chinese city by combining satellite data, meteorological information and a land use regression model. Environ. Pollut..

[91] Wang J., Zhang T., Fu B. (2016). A measure of spatial stratified heterogeneity. Ecol. Indic..

[92] Qiao P., Lei M., Guo G., Yang J., Zhou X., Chen T. (2017). Quantitative analysis of the factors influencing soil heavymetal lateral migration in rainfalls based on geographical detector software: A case study in Huanjiang County, China. Sustainability.

[93] Tang C., Yang C., Cai R.S., Ye H., Duan L., Zhang Z., Shi Z., Lin K., Song J., Huang X. (2019). Analysis of the relationship between electromagnetic radiation characteristics and urban functions in highly populated urban areas. Sci. Total Environ..

[94] He Q., Zhang M., Huang B. (2016). Spatio-temporal variation and impact factors analysis of satellite-based aerosol optical depth over China from 2002 to 2015. Atmos. Environ..

[95] Li L., Bakelants L., Solana C., Canters F., Kervyn M. (2018). Dating lava flows of tropical volcanoes by means of spatial modeling of vegetation recovery. Earth Surf. Process. Landf..

[96] Boiyo R., Kumar K.R., Zhao T., Bao Y. (2017). Climatological analysis of aerosol optical properties over East Africa observed from space-borne sensors during 2001–2015. Atmos. Environ..

[97] Goodale C., Aber J., Ollinger S. (1998). Mapping monthly precipitation, temperature, and solar radiation for Ireland with polynomial regression and a digital elevation model. Clim. Res..

[98] Li R., Wang Z., Cui L., Fu H., Zhang L., Kong L., Chen W., Chen J. (2019). Air pollution characteristics in China during 2015–2016: Spatiotemporal variations and key meteorological factors. Sci. Total Environ..

[99] Ng D.H.L., Li R., Raghavan S.V., Liong S.-Y. (2017). Investigating the relationship between aerosol optical depth and precipitation over Southeast Asia with relative humidity as an influencing factor. Sci. Rep..

